# Recent advances in solid-state NMR of zeolite catalysts

**DOI:** 10.1093/nsr/nwac155

**Published:** 2022-08-08

**Authors:** Weiyu Wang, Jun Xu, Feng Deng

**Affiliations:** National Center for Magnetic Resonance in Wuhan, State Key Laboratory of Magnetic Resonance and Atomic and Molecular Physics, Wuhan Institute of Physics and Mathematics, Innovation Academy for Precision Measurement Science and Technology, Chinese Academy of Sciences, Wuhan 430071, China; University of Chinese Academy of Sciences, Beijing 100049, China; National Center for Magnetic Resonance in Wuhan, State Key Laboratory of Magnetic Resonance and Atomic and Molecular Physics, Wuhan Institute of Physics and Mathematics, Innovation Academy for Precision Measurement Science and Technology, Chinese Academy of Sciences, Wuhan 430071, China; University of Chinese Academy of Sciences, Beijing 100049, China; National Center for Magnetic Resonance in Wuhan, State Key Laboratory of Magnetic Resonance and Atomic and Molecular Physics, Wuhan Institute of Physics and Mathematics, Innovation Academy for Precision Measurement Science and Technology, Chinese Academy of Sciences, Wuhan 430071, China; University of Chinese Academy of Sciences, Beijing 100049, China

**Keywords:** solid-state NMR, zeolites, characterization, active sites, reaction mechanism

## Abstract

Zeolites are important inorganic crystalline microporous materials with a broad range of applications in the areas of catalysis, ion exchange, and adsorption/separations. Solid-state nuclear magnetic resonance (NMR) spectroscopy has proven to be a powerful tool in the study of zeolites and relevant catalytic reactions because of its advantage in providing atomic-level insights into molecular structure and dynamic behavior. In this review, we provide a brief discussion on the recent progress in exploring framework structures, catalytically active sites and intermolecular interactions in zeolites and metal-containing ones by using various solid-state NMR methods. Advances in the mechanistic understanding of zeolite-catalysed reactions including methanol and ethanol conversions are presented as selected examples. Finally, we discuss the prospect of the solid-state NMR technique for its application in zeolites.

## Teaser

This review highlights the application of state-of-the-art solid-state NMR spectroscopy to zeolite catalysts and relevant catalytic reactions and discusses the prospect of solid-state NMR technique in the field of zeolites.

## INTRODUCTION

Zeolites have been widely applied in diverse areas including catalysis, ion exchange and separations in the chemical and petrochemical industry [1]. The crystalline framework of zeolites is constructed by the connecting of shared oxygen atoms of TO_4_ tetrahedra (typically AlO_4_ and SiO_4_). This composes a periodic and unique porous structure that can screen large molecules and only be accessed by molecules with a size equal to or smaller than the pore size, which contributes to the shape selectivity of zeolites. Meanwhile, the different valence electron shells of Al and Si tetrahedra contribute to a negative framework charge and when the exchangeable protons are introduced to balance the negative charge, it results in the formation of bridging hydroxyl groups (Si−OH−Al) which act as the Brønsted acidic sites (BASs) and gain the capability of catalysing traditional refinery reactions, including fluid catalytic cracking, alkylation and isomerization of hydrocarbons.

The Lewis acid sites (LASs) in zeolites are more complicated [2] and are often associated with either framework aluminum (FAL) species [3–6] or extra-framework Al (EFAL) species [7–10], the latter being generated by post-synthesis dealumination methods, such as steaming, calcination and acid or base leaching. Introducing heteroatoms such as titanium, tin, zinc, gallium and molybdenum as extra-framework species or into the framework is another approach for the generation of Lewis acidity. Besides, the exogenous metal species endow the zeolite with redox properties, broadening the catalytic application of zeolites in petrochemistry and biomass transformation [11,12]. In zeolites, BASs (or the hydroxyl group) and LASs not only exhibit their respective intrinsic acidic properties, but also can cooperate as synergistic sites, which leads to enhanced acid strength and higher catalytic performance in the catalytic process [13,14].

To rationally design zeolites with improved property, understanding the structure–activity relationship is a prerequisite. For zeolite structure characterization, X-ray diffraction (XRD), especially powder XRD, is routinely employed as an important analytic tool in zeolite science due to its capability of long-range ordering framework analysis. For short- to medium-range structure characterization, solid-state nuclear magnetic resonance (ssNMR) has been utilized as a sophisticated method with atomic-level resolution [15–23]. The local order of zeolite framework significantly influences the catalytic properties. For example, the Al organization in zeolites determines the distribution of acid sites. In a methanol-to-olefins reaction over ZSM-5, the samples with more acid sites in the channel intersections show higher selectivity to ethene and aromatics, while the samples with acid sites enriched in the sinusoidal and straight channels exhibit higher selectivity to propene and higher olefins [24]. For the ethanol-dehydration-to-diethyl-ether process, the associated reaction pathway (mediated by dimeric ethanol intermediate) is favored over ZSM-5 containing mainly isolated Al atoms at channel intersections, while the dissociative reaction pathway (mediated by the ethoxy group and accompanied by ethylene synthesis) is preferable over ZSM-5 with a dominating fraction of proximate Al atoms [25].

The structure information and molecular dynamic behavior can be reflected from multiple ssNMR parameters. Chemical shift is mostly used to distinguish and identify the diverse species coexisting in zeolites and provides detailed information on the local structure of the observed nuclei, owing to its high sensitivity to the surrounding electronic environment. For example, the acidic protons as well as hydroxyl groups including AlOH and SiOH can be distinguished by ^1^H NMR, and FALs and EFALs with different coordination states can be identified by ^27^Al chemical shifts. Besides, vital information can be extracted from the specific internuclear interactions, including dipolar and *J*-coupling interactions. Dipolar interaction, referred to as magnetic dipole–dipole interaction, is generated by the magnetic interaction between nuclei in close spatial proximity. This type of interaction contributes to line broadening and is generally eliminated by the magic angle spinning technique (MAS, spinning a sample at 54.736° with respect to the external magnetic field B_0_) to yield high-resolution spectra. On the other hand, since the strength of the dipolar interaction is inversely proportional to the cube of the intermolecular distance, it provides geometric information on molecules. Various advanced recoupling ssNMR techniques have been developed to recouple the dipolar interactions that are averaged out by MAS so as to measure internuclear distances or proximities [18,26]. The *J*-coupling interaction, referred to as a spin–spin coupling interaction, is present between two chemically bound atoms (usually no more than three chemical bonds). This interaction can be utilized by the *J*-coupling-based NMR technique to extract information on the chemical connectivity between certain atoms or structural constrains for solving the complex molecule structure. The magnitude of *J* coupling is rather small (about several to hundreds of Hz) compared to dipolar interactions with a strength of up to thousands of Hz, which makes the detection and utilization of *J*-coupling interaction more difficult [25]. These internuclear correlations can be probed by manipulating the nuclear spin interactions, technically achieved by applying advanced 2D ssNMR methods [26]. For example, the proximity/connectivity between different structural units in zeolites can be probed by ^29^Si–^29^Si and ^29^Si–^27^Al correlation spectra. The host–guest interaction is commonly present among many important processes in zeolites such as catalysis and crystallization. Abundant information about the local hybrid environment can be reflected by probing the interaction between the nuclei that represent the framework structure (host) and the confined species (guest) using ^1^H−^27^Al, ^1^H−^13^C, ^1^H−^29^Si and ^27^Al–^13^C double-resonance or 2D NMR. Zeolite-catalysed reactions involve intermolecular interactions between the adsorbed organic compounds in channels or cages [27]. 2D ^1^H–^13^C and ^13^C−^13^C correlation experiments are mostly applied to characterize these types of interactions, which offer molecular-level insights into the reaction mechanism in zeolite-catalysed reactions. Table 1 summarizes the zeolite-relevant topics discussed in this review and suitable ssNMR methods to be applied [28–43].

**Table 1.** Zeolite-relevant topics as studied by suitable ssNMR method.

Topic	Method	Nuclear spin interaction	Comments	Ref.
Framework connection	2D ^29^Si–^29^Si refocused INADEQUATE	*J* coupling	Identification of through-bond connectivity of framework Si species. ^29^Si isotropic enrichment is usually required.	[28]
Al distribution	2D ^27^Al–^27^Al DQ–SQ	Dipolar coupling	Identification of spatial proximity of Al atoms. Difficult to obtain quantitative distance information for multi-spin system.	[10,29]
Defect sites	2D ^1^H–^1^H DQ–SQ 2D ^1^H–^1^H TQ–SQ	Dipolar coupling	Identification of location and local structure of SiOH groups. TQ–SQ NMR suffers from a low efficiency in excitation of triple-quantum coherence.	[30,31]
Active site synergy	2D ^1^H–^1^H DQ–SQ	Dipolar coupling	Identification of spatial proximity between acidic protons and extra-framework Al species in dealuminated zeolites.	[9,32–34]
	1D ^1^H–{M} S-RESPDOR (M: metal)		Identification of spatial proximity between acidic protons and metal species. Quantitative determination is possible.	[35,36]
Host–guest interaction	1D and 2D ^13^C−^27^Al S-RESPDOR	Dipolar coupling	Identification of proximate guest organic species and Al site in zeolite framework. Structural determination of the surface organic species is possible.	[37–39]
Guest–guest interaction	2D ^1^H–^1^H PSD 2D ^13^C–^13^C PDSD	Dipolar coupling	Identification of organic compounds in close proximity. The intermolecular correlations could be influenced by the mobility and exchange process of the protons.	[40–43]

This present review is focused on the recent progress in the studies of zeolites by using the ssNMR technique. The application of ssNMR to investigate zeolite structures, intermolecular interactions and catalytic reaction mechanisms will be presented by selected examples from recent literature. The current limitation and prospects of the ssNMR technique and its application in zeolites will be discussed in the last part.

## ZEOLITE FRAMEWORK: Si, Al ATOMS AND SiOH DEFECTS

**Figure 1.**
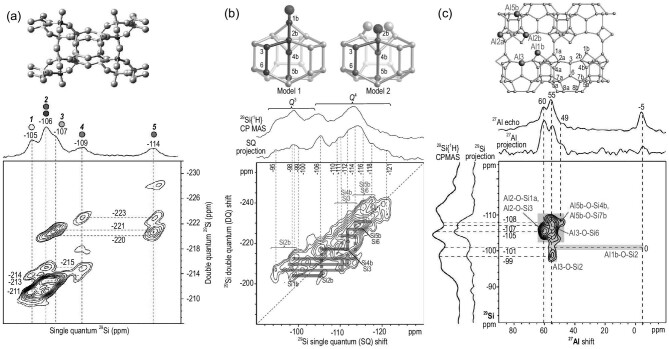
(a) 2D refocused INADEQUATE (*J*-mediated) ^29^Si{^29^Si} DQ NMR spectrum of as-synthesized zeolite ITW. Adapted from [28] with permission from the American Chemical Society. (b) DNP-enhanced 2D ^29^Si{^29^Si} *J*-mediated correlation spectrum of calcined Si-SSZ-70. Adapted from [47] with permission from the American Chemical Society. (c) Schematic diagram of the framework structure of Al-SSZ-70 (orange color indicates the T sites that are occupied by Al heteroatoms) and 2D ^27^Al{^29^Si} *J*-HMQC NMR spectrum of calcined Al-SSZ-70. Adapted from [59] with permission from Wiley-VCH.

The functions of zeolites are connected to their distinct framework structures. Therefore, determination of zeolite crystal structures is critical for evaluation of their potential in various applications. The zeolite structure solution usually depends on powder XRD due to the large single crystal not being available for most zeolites. However, the powder XRD approach alone is often challenging because of the structural complexity of zeolites. ssNMR has an advantage in revealing the short- to medium-range structure ordering in a zeolite framework, which provides complementary information for powder XRD. Silicon is one of the main elements in the zeolite framework. The Si atoms occupied in different positions in the zeolite framework can be identified based on ^29^Si chemical shifts and their interconnectivities can be reflected from 2D ^29^Si–^29^Si homonuclear correlation NMR spectroscopy [28,44–46]. In the recent work by Chmelka and co-workers, a *J*-mediated 2D refocused INADEQUATE ^29^Si{^29^Si} double-quantum (DQ) NMR method was applied to elucidate the ^29^Si–O–^29^Si site connectivities in the as-synthesized ITW zeolite [28]. At least five distinct resonances (labeled as 1–5) were observed in the single-pulse ^29^Si MAS NMR spectrum (Fig. 1a) and all can be correlated to different fully condensed Q^4^ Si sites with an approximate content ratio of 1:2:1:1:1. The cross-peak pairs shown in the 2D spectrum represented the covalent bonds between these Si sites. Si Site 1 (−105 ppm) exhibited correlation signals (−211 and −214 ppm) in the orthogonal dimension, which provided direct spectroscopic evidence of the connectivities with Si Sites 2 and 4, respectively. Similarly, the bond connectivities can be established between Site 2 and all other sites; Site 3 with Sites 2 and 5; Site 4 with Sites 1, 3 and 5; and Site 5 with Sites 2–5. Besides, the intensity of the cross peak provided additional information on bond connectivities. For example, the pair of correlated ^29^Si signals at (−105, −211) and (−106, −211) ppm, which were associated with the covalent bond between Sites 1 and 2, have nearly three times the magnitude of peak pairs at (−105, −214) and (−109, −214) ppm associated with the bond of Sites 1 and 4. This indicated that the Si Site 1 was bonded to three Site 2 and one Site 4. Considering the content of Site 2 was approximately two times larger than the rest of the Si sites, it was suggested that Si Site 2 was composed of two distinct Si sites that had approximate ^29^Si chemical shifts. Combining with XRD characterization, the sitings of the six distinct ^29^Si sites were identified. This work provides a versatile diffraction/NMR refinement technique for the zeolite structure solution, which may be not limited to the purely siliceous zeolites. Applying a sensitivity-enhanced technique further exploits the potential of ^29^Si–^29^Si correlation NMR spectroscopy in zeolite structure determination. For example, a structure analysis of the calcined high-silica zeolite SSZ-70 was made by using a 2D Dynamic Nuclear Polarization (DNP)-enhanced NMR technique [47]. Benefitting from the DNP-boosted sensitivity enhancement, the through-bond ^29^Si−O−^29^Si connectivity between neighboring Q^3^ and Q^4^ sites was clearly manifested and at least two distinct covalent linkages were established in the ^29^Si–^29^Si correlation NMR spectrum (in the blue line and red line in Fig. 1b).

Incorporation of Al atoms into a purely silica zeolite framework produces Brønsted acidity. The siting and distribution of Al atoms in the framework of zeolites dictate their acidic property and catalytic activity. Therefore, the understanding of the siting and distribution of Al atoms has important implications in zeolite modification and synthesis for tuning acid sites and the local environment in zeolite channels towards improved performance. However, the low concentration of Al atoms and abundant crystallographically inequivalent T sites in zeolites, particularly Si-rich frameworks, give rise to high variability of the Al siting and distribution, which makes their analysis challenging. Nevertheless, significant progress has been made in this area [48]. It was found that the framework Al siting and distribution are neither random nor controlled by statistical rules but rely on the synthesis conditions [49–52]. The NMR characterization of Al in different T sites is challenging owing to the quadrupolar property of Al nuclei, which generates strong quadrupolar interactions that broaden the ^27^Al NMR signal [53]. In the early study, successful differentiation of individual Al sites in zeolites by using 2D multiple-quantum (MQ) MAS experiments was reported [54–57]. At least 12 out of 24 framework T sites in ZSM-5 zeolite were determined by the combination of ^27^Al 3QMAS NMR and density functional theory method (DFT) calculations, allowing the Al siting to be partially resolved [51,57]. However, fully distinguishing Al atoms in different T sites is limited by the NMR spectral resolution. Taking advantage of the high magnetic field and low-temperature (<100 K) measurement conditions, ^27^Al NMR spectra with higher sensitivity and resolution can be obtained [58]. Most recently, Berkson *et al.* reported the utilization of through-bond 2D ^27^Al{^29^Si} *J*-correlation NMR spectra for the identification of Al site siting in a calcined Al-SSZ-70 zeolite [59]. According to the Lowenstein's rule, the Al–O–Al linkages are generally disfavored and typically absent at low Al content in zeolites. The position of Al species can be precisely specified by revealing the connectivities of Al atoms with certain Si sites. Four well-resolved ^27^Al signals were clearly observed (Fig. 1c): the signals at 60, 55 and 49 ppm were assigned to tetrahedrally coordinated ^27^Al species, while the −5 ppm signal came from octahedrally coordinated ^27^Al. The 2D ^27^Al{^29^Si} *J*-HMQC NMR spectrum revealed the bond connectivities of the distinct Al species with neighboring Si sites (red and blue areas). Since the ^29^Si chemical shifts of Si atoms sited at different T sites in SSZ-70 have been established, the occupations of Al atoms in T sites in the framework were determined according to the revealed ^27^Al–O–^29^Si connectivities. It was shown that 94% of the Al atoms were located at T2a/b, T3 or T1b sites on interlayer channel surfaces and only 6% are located at T5b sites on intralayer channel surfaces.

Besides the siting, Al distribution in the zeolite framework is another important factor significantly affecting catalytic activity because Al distribution governs the local density of acid sites and their properties. The distribution of Al sites is subject to the synthesis conditions [29]. Zeolite synthesis can be understood as an interplay among the structure-directing agents (SDAs), Si/Al sources and the other ions presented in the synthesis mixture. The existence of the OH^–^ anion under basic synthesis conditions would increase the polarization of SDA^+^ to balance two negative charges originating from AlO_4_^–^ tetrahedrons. Consequently, more Al atoms are concentrated near the cationic part of the SDA^+^ molecules, which eventually leads to a high fraction of Al pairs in the zeolite framework, whereas changing the OH^–^ anion to Cl^–^ or NO_3_^–^ would decrease the polarization of SDA^+^ and cause a decline in the concentration of Al pairs [52,60]. The existence of co-cations in the synthesis mixture also plays a critical role in altering the Al distribution, allowing the AlO_4_^–^ tetrahedrons to be balanced not solely by SDA cations. For example, the addition of Na^+^ can lead to an increase in the fraction of isolated Al among the framework in ZSM-5 preparation [61]. Part of the AlO_4_^–^ tetrahedrons are balanced by the Na^+^ cations instead of SDA ^+^, resulting in a high dispersion of Al atoms. The control of the Al distribution in zeolites would allow a catalyst with the desired catalytic functions to be obtained, which requires a deep analysis of the Al distribution in the zeolite framework. ^29^Si NMR is intensively used for the analysis of Si–Al connectivity in the Si(nAl,4-nSi) unit. This method prefers Al-rich zeolites, which however faces limitation for Si-rich samples. For example, the Si(2Al,2Si) and Si(3Si,1OH) units are often hard to be differentiated by ^29^Si NMR alone. 2D ^27^Al–^27^Al DQ NMR experiments can be used to establish the spatial correlation between Al atoms in close proximity in zeolites [10]. By using this method, various spatial correlations between framework Al and extra-framework Al sites were established in dealuminated Y zeolites, which provided insight into the dealumination mechanism of Al-rich zeolites. Alternatively, the Al correlation can be detected by using 2D ^1^H–^1^H DQ NMR experiments as the charge-balancing protons reside on the AlO_4_ unit [9]. Nevertheless, more information is expected on the Al distribution in zeolites, such as the exact location of the Al pairs in zeolite channels and rings, which represents a challenge for the NMR approach. To this end, UV–Vis–NIR spectroscopy of Co(II) ions is a powerful tool for the analysis of the Al distribution in well-calcined zeolites. The ion-exchanged Co^2+^ ions in dehydrated zeolites can be used as probes for the occurrence of Al pairs in the zeolite framework based on the fact that one exchanged Co^2+^ ion coordinates to a framework oxygen atom and is balanced by two framework AlO_4_ units, which produce different d–d transitions in the visible region reflecting the local coordination environment [49]. The spatial distribution of Al pairs [(Al–O–(Si–O)_1,2_–Al sequences] was found to be located in the same ring of the ZSM-5 framework, which correlates with the framework Al content. By analysing ^29^Si MAS NMR, fourier transform infrared (FTIR) and UV-Vis spectroscopy of the Co^2+^ ions probe, the recent work by Jiri *et al.* indicated that the concentration of Al pairs and single Al atoms in ZSM-5 can be tuned in a wide range by varying the composition of the synthesis gel [52].

Besides Al atoms, the T sites in the zeolite framework can be occupied by different heteroatoms, which endow the zeolites with distinct properties. The isomorphous substitution of boron atoms into MWW and MFI frameworks leads to isolated boron sites and their structures can be revealed by ^11^B NMR experiments. For example, 1D ^11^B MAS ssNMR and 2D ^11^B→^1^H D-RINEPT experiments demonstrated that the majority of boron species in B-substituted MWW existed as isolated BO_3_ units with a silanol group in the vicinity [62]. Borosilicate zeolites can convert to aluminosilicates through a post-synthesis process. Most recently, a site-preserved replacement of framework boron atoms by Al atoms was demonstrated on borosilicate zeolites SSZ-53, SSZ-55, SSZ-59 and SSZ-82 under hydrothermal treatment with an aqueous Al(NO_3_)_3_ solution [63]. 2D ^27^Al MQMAS NMR experiments jointly with DFT calculations indicated that Al occupied ordered positions in the four-ring chains of these zeolites. This observation shows potential to alter the catalytic property by the isomorphic substitution of framework atoms in zeolites.

**Figure 2.**
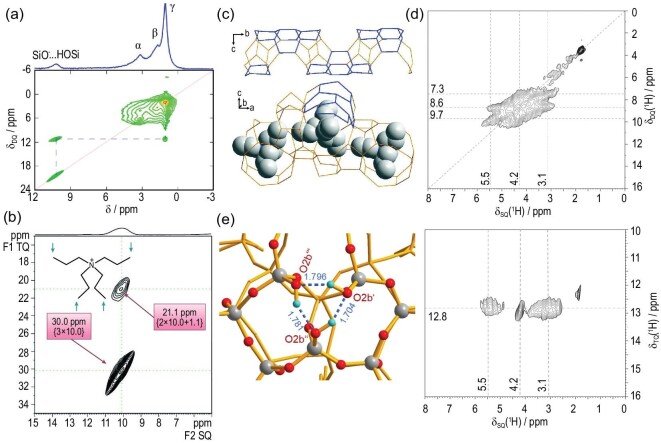
(a) 2D ^1^H DQ–SQ NMR spectrum of the as-synthesized TPA-silicalite-1. Adapted from [30] with permission from the American Chemical Society; (b) 2D ^1^H TQ–SQ NMR spectrum of the as-synthesized high-silica ZSM-5; (c) wire models of straight channel of ZSM-5 in different orientations and the bottom one shows the location of TPA^+^ cations. Adapted from [31] with permission from Wiley-VCH. (d) 2D ^1^H DQ–SQ MAS NMR and TQ–SQ MAS NMR spectra of calcined all-silica zeolite SSZ-70; (e) DFT-optimized structure of the silanol triad with a cyclic cluster model in SSZ-70. Adapted from [70] with permission from Wiley-VCH.

Zeolites are often not perfect crystalline materials since framework defects (i.e. various types of silanols) are generated during the synthesis or post-treatment. The zeolite defect is one of the decisive parameters affecting zeolite properties including hydrophilicity/hydrophobicity, stability and catalytic activity [64,65]. Besides, the defects in zeolites can act as anchoring positions for the incorporation of different heteroatoms (Mo, V, B, etc.) to create active sites. Elucidation of the defect sites is highly desirable to fine-tune zeolite properties and performance. Spectroscopic characterization techniques for zeolite defects are mainly XRD, UV–Vis, FTIR and ssNMR. For NMR experiments, 1D and 2D ^1^H NMR methods are mostly used due to the high abundance and sensitivity of ^1^H nuclei. Silanols and siloxy defects (SiO^–^) generally originate from the hydrolysis of Si−O−Si bridges (connectivity defects) and/or T sites dislodging (vacancy defects). Typically, the SiO^–^ groups are mostly formed in as-synthesized high-silica zeolites to compensate for the positive charge of the cationic SDAs confined in the zeolite channels. These defects are stabilized by a SiO^–^···HOSi hydrogen bond with nearby silanols and give rise to a characteristic ^1^H NMR signal at ∼10 ppm. As the SDAs have a certain location among zeolite frameworks, the location of defects can be determined by probing the SDA/framework interactions. The work by Eddy Dib *et al.* illustrated the application of 2D ^1^H DQ-SQ NMR experiments to reveal the location of defects among the as-synthesized tetrapropylammonium (TPA) directed silicalite-1 structure [30]. In the correlation NMR spectrum (Fig. 2a), the off-diagonal cross peaks were clearly observed between framework defects (10.2 ppm) and terminal methyl groups in TPA^+^ (H_γ_ at 1.0 ppm), indicating that the defects were located in the vicinity of methyl groups. Since SDA terminal methyl groups were known to be in the middle of zeolite channels, it can be concluded that that the defects were mostly located in the two zeolite channels rather than at the intersection of channels. Moreover, it was further revealed that the defects would preferentially locate in the sinusoidal channels when non-symmetric SDAs were used [66].

The charged SiO^–^ group generated from vacancy or connectivity defects in the zeolite framework could form hydrogen bonds with two or three silanols and multiple models have been proposed [67,68]. Triple-quantum/single-quantum (TQ–SQ) NMR spectroscopy has been applied to gain more insights into the local structure of defect sites. Similar to the DQ–SQ spectroscopy that reveals the proximity between at least two protons, TQ–SQ spectroscopy is able to probe the correlation among a cluster of at least three protons [69] and thus more structure constrains can be provided. For example, the ^1^H TQ–SQ spectrum of high-silica ZSM-5 synthesized with TPA cations confirmed that the defect sites were constituted of a set of three SiO^–^···HOSi hydrogen bonds in proximity as reflected by a cross peak at 30 ppm (3 × 10 ppm) in the TQ dimension [31] (Fig. 2b). Similar results were observed on high-silica ZSM-12 and high-silica SSZ-74 zeolites. Additionally, the defects in the straight channel of ZSM-5 seemed to be selectively located in the motif of four six-rings, as the methyl groups of the TPA^+^ cation residual were close to these motifs (Fig. 2c). This can be explained by the steric hindrance that these connectivity defects cannot be easily formed in the four- or five-rings. It should be noted that the formation of defects is very specific and can be easily altered by the synthesis conditions and zeolite properties. For silicalite-1 zeolite, the defect sites were demonstrated to be constituted of silanols pairs instead of a triad model according to the combination of DQ–SQ and TQ–SQ NMR spectroscopy [30]. The local structure was further described as a cluster of two SiOH and two SiO^–^ groups with two SDA methyl groups in close proximity. However, such a configuration was questioned by some researchers as it involved two like charges next to each other, causing Coulomb repulsion [31]. Most recently, another pair model defect was claimed on the as-synthesized SSZ-70, generated by two silanol groups hydrogen-bonding with one charged SiO^–^ moiety [70]. These results clearly demonstrate that the defects could be represented by either a pair or triad model, indicating the complexity of the zeolite framework.

Calcination treatment is routinely applied for the removal of the SDAs in as-synthesized zeolites to form the final porous materials. After removing the SDAs, the demand for the charge compensation of the zeolite framework is dismissed and a decrease in the number of defects can be expected. However, some silanol groups can remain constant after the calcination treatment. Isolated silanols produce ^1^H NMR signals of 1.2–2.0 ppm, while hydrogen-bonded silanols are in the range of 2.6–8.4 ppm. These signals often suffer a significant overlapping in the ^1^H NMR spectra, which has attracted considerable attention. In the recent work of Dib *et al.*, the silanol species in pure silica MFI-type zeolites were analysed by ^1^H NMR and infrared (IR) spectroscopy coupled with DFT calculations [71]. Four types of silanols with different extents of participation in the complex hydrogen-bonded silanol networks were identified. The correlations between critical geometrical parameters and spectral characteristics have been revealed: increasing the strength of the hydrogen bond would lead to decreased hydrogen-bond length, elongation of the O–H bond, as well as increased chemical shift in ^1^H NMR and decreased O–H vibrational frequency in FTIR. The flexibility of the zeolite fragment was found as the key factor determining the formation and strength of hydrogen-bonded silanols. Generally, for the hydrogen-bonded silanols, SiOH pairs are often observed [72], while the SiOH triad or SiOH tetrad is not expected as they would be easily condensed and show lower stability [73]. By using 2D DQ–SQ and TQ–SQ NMR spectroscopy, a stable silanol triad was determined in the calcinated SSZ-70 zeolites [70]. Three distinct silanol sites with ^1^H signals at 3.1, 4.2 and 5.5 ppm can be clearly identified (Fig. 2d). The cross-peak pairs at 7.3 (3.1 + 4.2), 8.6 (3.1 + 5.5) and 9.7 (4.2 + 5.5) ppm in the DQ dimension indicated the proximity among these silanol sites, corresponding to a SiOH triad structure. Moreover, this cluster of three silanols was evidently observed in the TQ–SQ spectrum, reflected by a series of cross peaks shared with a same value of 12.8 (3.1 + 4.2 + 5.5) ppm in the TQ dimension. The structures of this silanol triad on calcined SSZ-70 were theoretically confirmed with the assistance of DFT calculations. A cyclic triad and an open triad with one OH-bond bridging another Si–O–Si oxygen atom across an adjacent 5-ring were compared in terms of ^1^H chemical shifts and stabilization energy. The silanol triad nest with the cyclic cluster model was found to best fit the experiment data (Fig. 2e).

## ACTIVE SITES ON ZEOLITES

**Figure 3.**
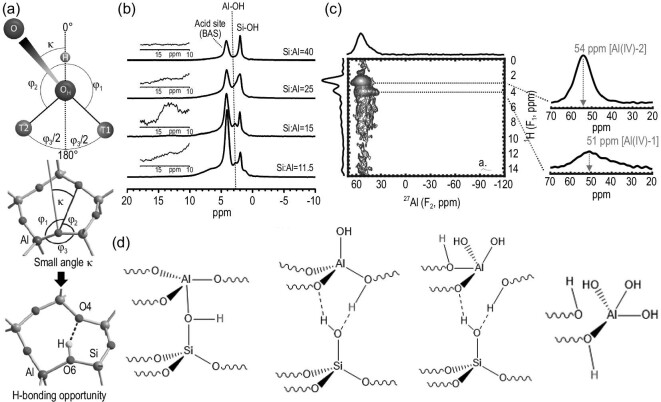
(a) Definitions of the atoms and angles involved in finding the κ values for evaluating the suitability of an oxygen donor (OH) and acceptor (O) pair. Adapted from [75] with permission from the American Chemical Society. (b) ^1^H MAS NMR spectra acquired at room temperature of dehydrated H-ZSM-5 prepared under vacuum heating. Adapted from [79] with permission from the American Chemical Society. (c) 2D ^27^Al{^1^H} D-HMQC NMR spectrum of dehydrated H-ZSM-5 at 35.2 T and (d) schematic of the well-known BAS in the zeolite lattice, as well as intermediate structures formed via the attack of one, two and three water molecules at the BAS. Adapted from [80] with permission from the American Chemical Society.

The BASs together with other hydroxyls including SiOH groups and extra-framework AlOH groups on zeolites constitute a complex proton environment in zeolites. The structural characterization of these hydroxyls is very important for understanding their properties. The spectroscopic information can be faithfully obtained from ^1^H MAS NMR [74], with characteristic chemical shift ranges at 3.6–5.2, 0.6–3.6 and 1.2–2.2 ppm for Brønsted acidic proton, extra-framework AlOH groups and isolated SiOH groups, respectively. By measuring the integrated area of the corresponding ^1^H signals, a quantitative analysis of hydroxyl groups in zeolites can be obtained. There is a consensus that the BASs in defect-free zeolites with high Si/Al ratios have identical acid strength. In the recent work of Koller and co-workers [75], a specific BAS forming a hydrogen bond with a framework O atom in the vicinity was proposed and referred to as perturbed BAS to distinguish it from the conventional BAS (unperturbed BAS). Generation of such BASs required a certain geometric orientation between the BAS-incorporated O atom and the potential hydrogen-bond acceptor O atom, and a low angle (κ) between the possible O−O pairs and the O−H axis in the BAS was favored (Fig. 3a). Nearly one-third of the oxygen atoms among 27 zeolite topologies can allow such a perturbed BAS formation. The debated ^1^H NMR signal at near 6 ppm in H-ZSM-5 was attributed to this perturbed BAS. Note that this chemical shift range may overlap with the signals of hydrogen-bonded SiOH defects and other surface hydroxyls in the ^1^H NMR spectra. 2D correlation NMR enables a clear distinction of various proton species [76]. Hydrogen-bonded SiOH defects with paired hydroxyls were precisely identified using ^1^H DQ–SQ NMR while the acidic hydroxyls on BASs can be determined using ^1^H{^27^Al} REAPDOR NMR. Taking these experiments together, the ^1^H signals at 3.3 and 4.0 ppm were assigned to hydrogen-bonded SiOH groups and the signals at 4 and 6 ppm were assigned to unperturbed and hydrogen-bonded BASs on high-silica H-ZSM-5, respectively [76]. FTIR is often used complementarily to NMR spectroscopy in the characterization of OH groups in zeolites [77]. The work by Chizallet *et al.* theoretically predicted the signals of possible hydroxyls on the external of H-ZSM-5. By cross-checking the results of FTIR and 2D ^1^H DQ–SQ NMR experiments, an unambiguous identification of the surface hydroxyl groups and their proximities was achieved [78].

The work by Chen *et al.* reported the existence of a new BAS associated with distinct tetrahedral aluminum atoms in H-ZSM-5, which exhibited superior catalytic behavior in benzene hydride-transfer and n-hexane cracking reactions [79,80]. The ^1^H MAS NMR spectra show that in addition to the generally recognized signals at 4.2, 2.8 and 2 ppm that are ascribed to BASs, EFAL-associated AlOH and SiOH, respectively, a broad new signal at 12–15 ppm was observed on zeolites with lower Si/Al ratios [79] (Fig. 3b). It was confirmed by isotopic H/D exchange experiments that such species and AlOH can undergo proton transfer with a deuterated probe molecule, representing their BASs acidic nature. Ultra-high magnetic field (35.2 Tesla) ^27^Al{^1^H} DQ–SQ correlation NMR in conjunction with ^1^H DQ–SQ experiments revealed that these species were correlated with a new tetrahedrally coordinated site [Al (IV)-2], which has an increased chemical shift and distinct quadrupolar parameters relative to the conventional BASs [80] (Fig. 3c). The possible configurations of the distinct BASs were examined using DFT calculations. The calculated quadrupolar parameters and chemical shifts of different Al sites agreed with the experimental results and supported the proposed partially hydrolysed acidic sites (Fig. 3d).

**Figure 4.**
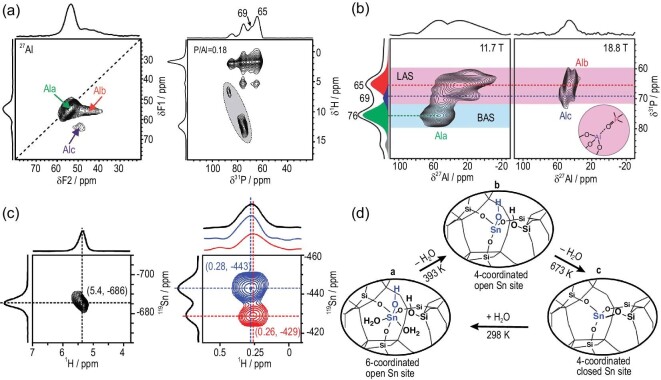
(a) 2D ^27^Al 3QMAS NMR and 2D ^31^P{^1^H} CP HETCOR MAS NMR spectra of dehydrated H-ZSM-5 with a low TMPO loading; (b) 2D ^31^P{^27^Al} PT-D-HMQC MAS NMR spectrum recorded at 11.7 T and 2D ^27^Al{^31^P} PT-D-HMQC MAS NMR spectrum recorded at 18.8 T of the dehydrated H-ZSM-5 with a medium TMPO loading. Adapted from [83] with permission from the Royal Society of Chemistry. (c) 2D ^1^H–^119^Sn HMQC MAS NMR spectra of ^119^Sn-β without dehydration and dehydrated at 393 K with ^119^Sn decoupling; (d) schematic of interconversion between open and closed Sn sites in Sn-β zeolite. Adapted from [97] with permission from Springer Nature.

Apart from BASs, LASs are another important factor that influences zeolite acidity and catalytic performance. Typically, one of the four Al–O–Si bonds of a fully condensed Q_4_ Al T-atom in a zeolite framework would firstly break up under dealumination conditions, leading to the formation of a tri-coordinated FAL [Al(OSi)_3_] and a framework silanol group (SiOH) in the vicinity. Upon the continuous breaking of the Al–O–Si bonds, the partially bonded tri-coordinated FAL would be eventually dislodged from the framework and turn into an EFAL species [81]. Compared with the substantial studies of the Lewis acid properties of EFAL [82,83], the spectroscopic understanding of tri-coordinated FAL as a framework LAS is considerably limited [82,84]. One of the main difficulties lies in the observation and differentiation of such species. In the ^27^Al NMR characterization of the tri-coordinated FAL, the reduced number of oxygen groups enhances the distortion of Al nuclei and results in remarkable signal broadening (C_Q_ > 30 MHz), rendering the tri-coordinated FAL species ‘NMR-invisible’. Combined with ^27^Al{^1^H} REDOR and ^27^Al 3Q MAS NMR, Brus *et al.* identified a broad ^27^Al NMR signal (59–62 ppm, C_Q_ = 5 MHz, η = 0.3–0.4) on different hydrated zeolites and correlated this signal with the hydrous tri-coordinated FAL species, thus implying the possible existence of tri-coordinated FAL species in dehydrated zeolites [82]. The recent work of Xin *et al.* provided direct experimental evidence from NMR spectroscopy of the generation of two distinct tri-coordinated FAL species in dehydrated H-ZSM-5 zeolites [83]. Trimethylphosphine oxide (TMPO) that is routinely applied as a base probe molecule for the characterization of acid sites in zeolites [85–87] was used to interact with the ‘NMR-invisible’ tri-coordinated FAL sites and turned them into NMR-observable distorted tetrahedral FAL. In the 2D ^27^Al 3QMAS NMR spectrum of dehydrated H-ZSM-5 with adsorbed TMPO (Fig. 4a), three distinct Al sites were clearly identified (Al_a_, Al_b_, Al_c_). 2D ^31^P{^1^H} CP HETCOR MAS NMR spectrum confirmed that both BASs and LASs were present on the sample (Fig. 4a), while 2D ^31^P{^27^Al} Population Transfer Heteronuclear Dipolar-mediated Multiple Quantum Correlation (PT-HMQC) spectra indicated that the ^31^P signals at 65 and 69 ppm were generated from TMPO molecules directly adsorbed on the FAL-associated LAS species Al_b_ and Al_c_, respectively (Fig. 4b). This unambiguously revealed that these Al species were tri-coordinated FAL sites bound with TMPO molecules.

The incorporation of metal atoms (Sn, Ti, Ga, Cu, etc.) into zeolites is an important way to generate LASs [88–92]. These metal species can be introduced as counter ions to compensate for the negative charges generated from the AlO_4_ units in zeolites. The metal-containing zeolites significantly distinguish from the pure silicon or proton counterparts in many catalytic processes such as alkanes conversion and biomass transformations. The determination of metal sites by NMR experiments is challenging due to the complexity of their structures and similar local environments in the framework. Since the active metals in Lewis acid zeolites often feature low loading (usually <2%) and low natural abundance (e.g. 8.6% for NMR active ^119^Sn), their direct NMR observations remain challenging [93–95]. ^31^P MAS NMR of TMPO adsorption was utilized to distinguish different active Sn sites in Sn-beta [96]. Sn sites with different coordinated states correlated with distinct ^31^P NMR signals. In the recent work of Qi *et al.*, two types of open Sn sites were clearly identified on ^119^Sn-enriched Sn-β by using proton-detected 2D ^1^H {^119^Sn} correlation NMR spectroscopy [97]. On the hydrated Sn-β, only water bonded 6-coordinated Sn sites were observed at (5.4, −686) ppm (Fig. 4c), since only the protons interacting with Sn species appeared in the ^1^H–^119^Sn dipolar interaction mediated spectrum. On the dehydrated sample, two correlation peaks [(0.28, −443) and (0.26, −429) ppm] provided clear evidence of the existence of two distinct open Sn−OH sites in the Sn-β framework. The interconversion can occur between open and closed Sn sites in Sn-β zeolites during the dehydration and hydration process (Fig. 4d).

**Figure 5.**
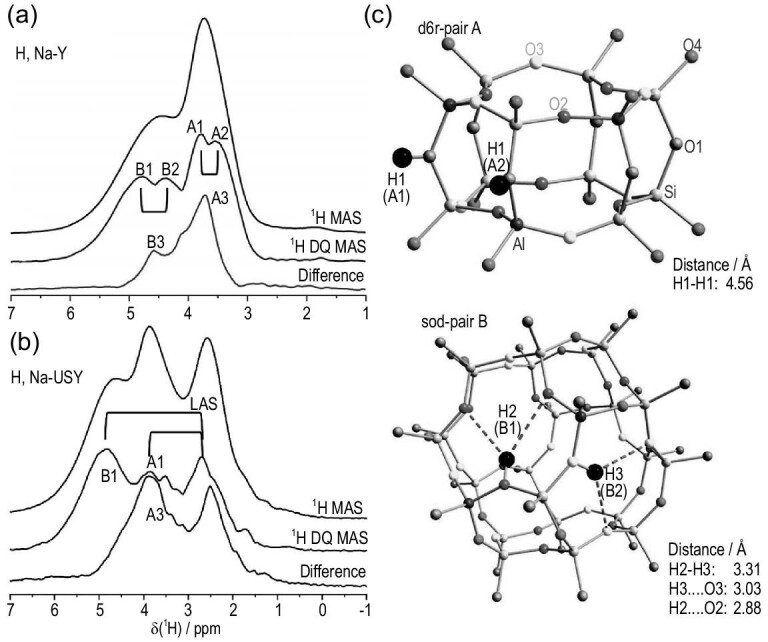
(a) Separation of paired and isolated BAS in dehydrated H, Na–Y zeolite and (b) detection of paired BAS and LAS in dehydrated H, Na-USY by ^1^H MAS, DQ NMR (projection of SQ dimension of the 2D experiment) and difference spectra; (c) proposed proton pairings for the BAS–BAS pairs in zeolite H, Na–Y. Adapted from [98] with permission from Wiley-VCH.

Different types of active sites can be simultaneously present in zeolites. The synergistic effect of active sites in zeolites is generally observed among spatially proximate BASs and LASs, usually resulting in enhanced catalytic performance. The determination of synergistic sites is a prerequisite for uncovering the synergistic mechanism in catalytic reactions. In dealuminated zeolites, the synergistic active sites are mainly constituted by the adjacent BASs and EFAL species (acting as LASs). 2D ^1^H–^1^H and ^27^Al–^27^Al DQ MAS NMR methods capable of detecting internuclear coupling between proximate nuclei are extensively utilized to probe the spatial proximity/interaction between different acid sites. As an example, Brønsted/Lewis acid synergy over dealuminated HY zeolite was revealed by 2D ^1^H–^1^H DQ NMR, which established the correlation between the hydroxyls in BASs and EFAL [9,32]. 2D ^27^Al–^27^Al DQ MAS NMR was also employed to detect the proximity between Al atoms in BASs and EFAL species in dealuminated zeolites [10,33,34], in which the close proximities among four-coordinate FAL (BAS), five- and six-coordinate EFAL species (LASs) were identified. Most recently, 2D ^1^H–^1^H DQ MAS NMR has been applied to study the ultra-stabilization process of zeolite Y [98]. The combination of ^1^H MAS NMR and 2D ^1^H–^1^H DQ MAS NMR clearly revealed the Brønsted–Brønsted acid pairs (A1/A2 in super cage and B1/B2 in sodalite cage) and isolated BASs (A3/B3) in H, Na–Y (Fig. 5a). After the ultra-stabilization process (dealumination), a conversion of the BAS–BAS pairs into BAS–LAS pairs occurred as reflected by the newly formed pairs of BAS and LAS (B1/LAS and A1/LAS) (Fig. 5b and c).

**Figure 6.**
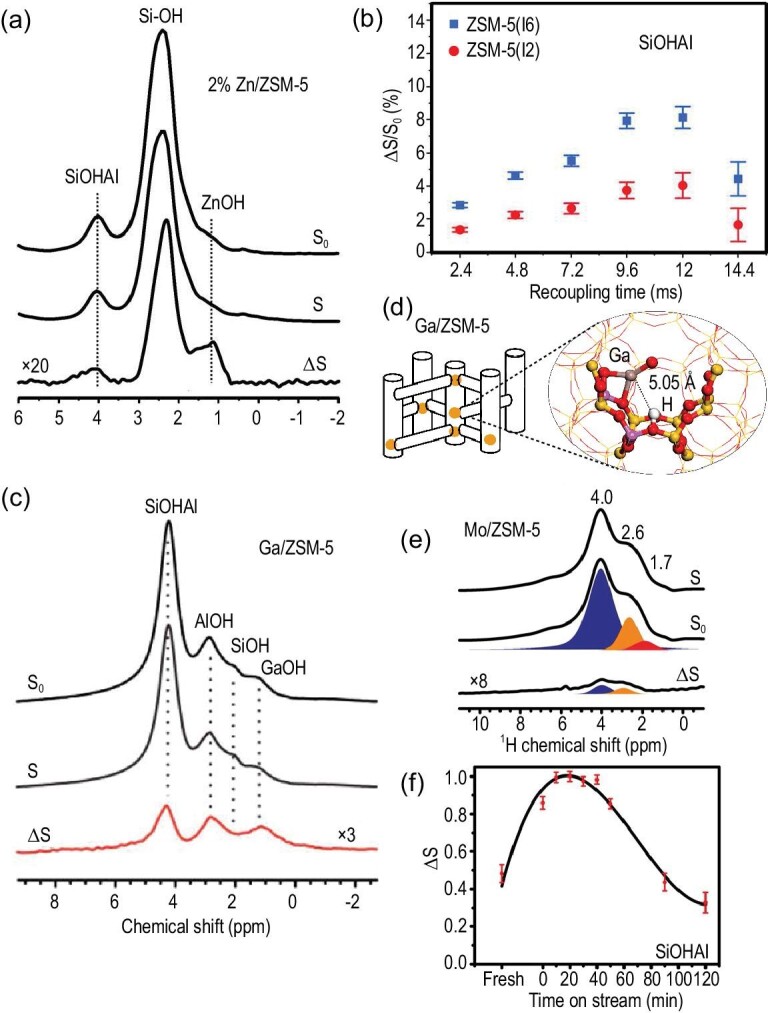
(a) ^1^H–^67^Zn S-RESPDOR NMR spectra recorded at 18.8 T of Zn/ZSM-5 (2 wt.%, ^67^Zn-enriched) with a recoupling time of 9.6 ms; (b) ΔS/S_0_ signal (Brønsted acid site) fraction versus recoupling time. Adapted from [103] with permission from Wiley-VCH. (c) ^1^H–^71^Ga S-RESPDOR NMR spectra of Ga/ZSM-5 with a recoupling time of 12 ms; (d) model of proximate Ga species and BAS in ZSM-5 channel. Adapted from [35] with permission from the American Chemical Society. (e) ^1^H–^95^Mo S-RESPDOR NMR spectra acquired at 18.8 T of fresh Mo/ZSM-5 (5 wt.%, ^95^Mo enriched) with a recoupling time of 5.12 ms; (f) normalized ΔS of Brønsted acid proton versus MDA reaction time. ΔS = S_0_ – S. Adapted from [36] with permission from Wiley-VCH.

In metal-containing zeolites, the synergistic effect is generated by the spatial interaction between the intrinsic BASs and the introduced metal species (LASs). The direct evidence of this effect can be obtained by detecting the spatial proximity between acidic protons and metal species using the double-resonance NMR method. Owing to the high sensitivity of ^1^H nuclei, proton-detected ^1^H–X (X: metal) double-resonance NMR offers a versatile approach to reveal the structure of synergistic sites in metal-containing zeolites [17]. Zinc-modified zeolites exhibit distinct activity in the transformation of light alkanes to oxygenates and aromatics, which has attracted much research interest in their structural and property analysis [99–102]. The spatial proximity between Brønsted acidic protons and zinc species on the Zn/ZSM-5 catalyst was probed using symmetry-based RESPDOR (S-RESPDOR) NMR [103]. A significant signal dephasing was observed on the SiOHAl group (Fig. 6a), indicating a spatial proximity between the acidic protons and the introduced Zn species. The ^1^H–^67^Zn internuclear distance (2.70–3.34 Å) between the Brønsted acidic proton and Zn^2+^ was extracted from the build-up curves (Fig. 6b). Moreover, a quantitative determination of the synergistic active sites can be achieved using the S-RESPDOR NMR experiment. Further ^1^H MAS NMR analysis demonstrates that the synergistic effect largely enhances the Brønsted acid strength as well as the methane H/D exchange activity of Zn-modified zeolites. Analogously, ^1^H–^71^Ga S-RESPDOR NMR was used to detect the spatial proximity between Ga species and BAS on Ga-modified ZSM-5 zeolites [35] (Fig. 6c). The internuclear distance between the BAS–Ga pair experimentally measured using S-RESPDOR NMR was 5.05 Å, which is close to the ^1^H–^1^H distance (∼4.50 Å) between the neighboring Brønsted acidic protons in the six-membered ring of ZSM-5 as determined by the 2D ^1^H–^1^H DQ MAS NMR experiment [33]. This suggests that the BAS–Ga pair was formed by substitution of one proton from the Brønsted acidic pair by the Ga species (Fig. 6d). The content of the synergistic BAS–Ga pair was found to be closely related to the aromatic selectivity in the methanol-to-aromatics reaction.

The active sites in zeolites may undergo change during catalytic reactions. The dynamic synergistic active sites in Mo/ZSM-5 were monitored by using ^1^H–^95^Mo S-RESPDOR NMR spectroscopy [36]. The spatial proximity between BASs and active Mo species was observed on fresh Mo/ZSM-5 and the samples reacted for different times in the methane dehydroaromatization (MDA) reaction (Fig. 6e). The ^1^H–^95^Mo S-RESPDOR dephasing effect (ΔS) as a function of the reacting time demonstrated the evolution of the acidic proton–Mo synergic sites (Fig. 6f). The significant increase in ΔS in the initial stage of the MDA reaction (0–30 min) indicated the migration of Mo species from the external surface into zeolite channels to form more proximate ^1^H–^95^Mo sites; the subsequent decreasing of ΔS at longer reaction time corresponded to an increasing distance between the ^1^H–^95^Mo pairs, which suggested the detaching of active Mo species from zeolite channels to the external surface. The evolution of the ^1^H–^95^Mo pairs can be correlated with the catalytic performance of the Mo/ZSM-5 catalyst in the MDA reaction.

## HOST–GUEST AND GUEST–GUEST INTERACTIONS IN ZEOLITES

**Figure 7.**
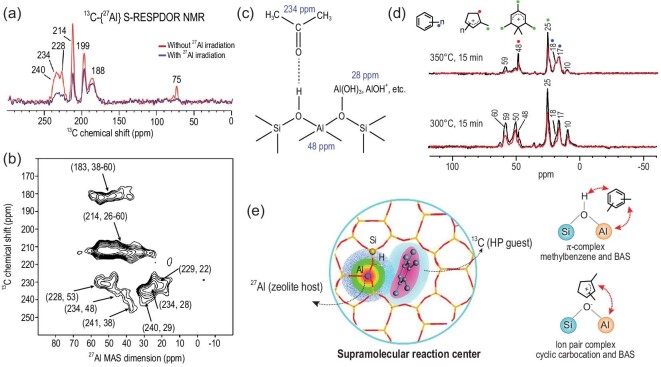
(a) ^13^C–^27^Al S-RESPDOR NMR spectra of 2–^13^C-acetone loaded on dealuminated HY zeolite; (b) 2D ^27^Al–^13^C D-HMQC NMR spectrum of 2–^13^C-acetone loaded on dealuminated HY zeolite acquired at 18.8 T; (c) schematic of 2–^13^C-acetone adsorbed on the BAS on dealuminated HY. Adapted from [37] with permission from the American Chemical Society. (d) ^13^C–^27^Al S-RESPDOR NMR spectra of trapped organic compounds obtained from MTO reaction over H-ZSM-5 at 300°C for 15 min; (e) models for the SRC in zeolite channels. Adapted from [38] with permission from Wiley-VCH.

In zeolites, the intermolecular interactions including host–guest and guest–guest interactions play critical roles in zeolite synthesis, adsorption/desorption and catalytic reactions. Host–guest interactions involve a zeolite framework host and an adsorbed molecule guest. The analysis of various host–guest interactions in zeolites is important for understanding the properties of zeolites and the catalytic process from the point of view of both active sites and reactant molecules. The active sites in the zeolites framework are often associated with Al species, while carbon is the main element that constitutes the molecular backbone of adsorbed organics. Therefore, the direct and desirable way to probe the host–guest interactions in zeolites is the application of the ^13^C−^27^Al double-resonance NMR technique [104–107]. Acetone is a base probe molecule capable of detecting the surface acidity of solid acids [108,109]. The host–guest interaction between adsorbed acetone and dealuminated HY was revealed by using 1D and 2D ^27^Al–^13^C correlation NMR spectra [37] (Fig. 7a and b). The acetone molecule adsorbed on the BAS simultaneously had a close proximity to EFAL, which evidenced the spatial proximity of a FAL–EFAL species on dealuminated HY (Fig. 7c). In zeolite-catalysed methanol conversion, supramolecular reaction centers (SRCs) were supposed to be formed by complexing zeolite framework Brønsted acid/base sites with retained hydrocarbon pool (HP) species such as cyclopentenyl cations, methylbenzenes and benzenium ions [110]. Direct experimental evidence for the existence of this reaction center was provided by using ^13^C–^27^Al S-RESPDOR NMR experiments [38]. Spatial proximity/interaction between the formed HP species and framework acid sites was evident over H-ZSM-5 in the methanol-to-olefins (MTO) reaction (Fig. 7d). The trapped methylbenzenes and cyclic carbocations HP species interacted with the BAS forming a π-complex structure and an ion-pair complex, respectively (Fig. 7e). It was found that a stronger interaction corresponded to a higher reactivity of the SRC in the MTO reaction. The similar SRC was confirmed over other zeolite topologies such as H-SSZ-13 (CHA-type) and H-MOR (12-membered ring) zeolites, suggesting its important role in the prevailing HP process in the MTO reaction over the zeolites [111].

**Figure 8.**
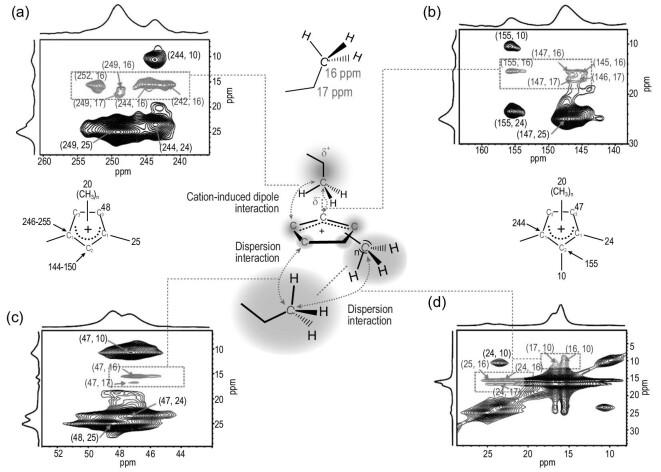
(a–d) 2D ^13^C–^13^C PDSD NMR spectra of ^13^C-labeled propane adsorbed on cyclopentenyl cation containing H-ZSM-5. The intra- and intermolecular correlation peaks are colored in black and blue, respectively. Adapted from [43] with permission from Wiley-VCH.

Guest–guest interactions are presented among the adsorbed organic molecules that are trapped in zeolite channels and cavities. The detection of such interactions is challenging because the involved organic molecules are weakly coupled via non-covalent interactions. Most recently, high-resolution proton spin diffusion (PSD) NMR spectroscopy in combination with DFT calculations and molecular simulations was utilized to probe the aggregation states of chiral SDAs [(1R,2S)-ephedrine] as monomers or dimers confined within the MgAPO-5 materials. Increasing the spin diffusion time leads to a decrease in the intensity of the diagonal peaks and an increase in that of off-diagonal cross peaks in the ^1^H PSD NMR spectra. By estimating the evolution of the diagonal peak with the spin diffusion time, the average distances between the aromatic rings and their closest protons in both aggregation states were determined [40]. The non-covalent interactions play an important role in catalysis as they stabilize both transition states and active intermediates. Therefore elucidation of the catalytic reaction process can be aided by the analysis of the non-covalent interactions among various confined organic molecules. 2D ^13^C–^13^C Proton-Driven Spin Diffusion (PDSD) correlation NMR spectroscopy has a distinct advantage in characterizing intermolecular interactions [41]. Cyclopentenyl cations are important HP intermediates in the MTO reaction and participate in hydrocarbon formation and catalyst deactivation. By using 2D ^13^C–^13^C PDSD spectroscopy, the evolution of the intermolecular π-interactions between methylbenzenes and cyclopentenyl cations was revealed in H-SSZ-13 and H-ZSM-5 zeolites in the MTO reaction [42]. The formation of naphthalene as a precursor to coke species was promoted by the observed intermolecular interactions among the bulky species, which was correlated with the deactivation process. The overall positive electrostatic potential enables the cyclopentenyl cations to be an electron receptor to molecules with an electron-rich entity such as anion, lone-pair electrons, π electrons, etc. Additionally, the attached alkyl groups of cyclopentenyl cations can produce van der Waals interactions with organic compounds. Therefore, various intermolecular non-covalent interactions could be formed between cyclopentenyl cations and hydrocarbons during the MTO reactions over zeolites [43]. Taking propane as an example, multiple non-covalent interactions were identified between propane and cyclopentenyl cations in the 2D ^13^C–^13^C PDSD NMR spectra of ZSM-5 (Fig. 8). Propane was inductively polarized and generated partially negative charges that were attracted by cyclopentenyl cations, which produced a cation-induced dipole interaction and thus the intermolecular proximity (Fig. 8a and b). The induced dispersion force allowed the observation of the additional intermolecular interactions between propane and weakly-polar groups of cyclopentenyl cations including methyl and methylene (Fig. 8c and d). The strong attractive forces between cyclopentenyl cations and methanol as well as ethene were also observed, revealing the existence of cation–dipole interaction and cation–π interaction, respectively. These carbocation-induced non-covalent interactions were demonstrated to promote methanol reaction and transformation of the intermediate products such as alkanes and olefins in the MTO reaction.

**Figure 9.**
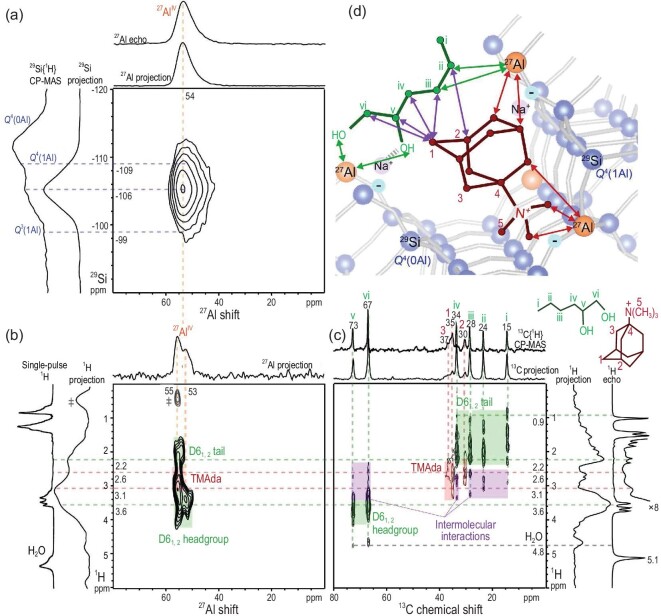
(a) 2D ^27^Al{^29^Si} *J*-mediated NMR correlation spectrum; (b) 2D ^27^Al{^1^H} HETCOR NMR spectrum; (c) 2D ^13^C{^1^H} HETCOR NMR spectrum of the as-synthesized HOU-4 zeolite and (d) schematic diagram of framework-OSDA and intermolecular interactions established by 2D NMR spectroscopy. Adapted from [39] with permission from the American Chemical Society.

A collection of detailed information about the host–guest and guest–guest interactions can provide more structure constraints for the elucidation of a complex structure. One example is zeolite syntheses using organic structure-directing agents (OSDAs), which involve various inorganic–organic and inorganic–inorganic interactions in crystallization. 2D correlation NMR characterization techniques were utilized to examine the role of dual OSDAs *N*, *N*, *N*-trimethyl-1,1-adamantammonium (TMAda^+^) and 1,2-hexanediol (D6_1,2_) in the formation of the HOU-4 crystal (mordenite framework type) [39]. In the as-made HOU-4, 2D ^27^Al{^29^Si} *J*-mediated NMR correlation experiment probed the host–guest interactions between OSDA molecules and zeolite frameworks and revealed that the covalent Al–O–Si units composed of framework Al atoms bonded to fully (Q4) or partially (Q3) cross-linked Si atoms (Fig. 9a). Two types of Al sites (55 and 53 ppm) were distinguished in the 2D ^27^Al{^1^H} HETCOR NMR spectrum: one spatially interacted with TMAda^+^ within the 12-ring channels and the other one was close to D6_1,2_ coordinated to Na^+^ cations within the 8-ring pockets (Fig. 9b). Moreover, a close mutual proximity between the two SDA molecules was clearly identified using 2D ^13^C{^1^H} HETCOR NMR (Fig. 9c), which evidenced the cooperative role of the SDAs in directing the hydrothermal crystallization of HOU-4 and Al distribution within zeolite channels (Fig. 9d). A further combination of microscopy, modeling and 2D NMR techniques provided deep insights for understanding how the SDAs tailor the crystallization and physicochemical properties of zeolites.

## CATALYTIC MECHANISMS REVEALED BY *INSITU*/*OPERANDO*ssNMR

Understanding the catalytic reaction process at the molecule level is essential for the establishment of the structure–activity relationship towards rational catalyst design for particular reactions. Much effort has been focused on the observation and structural determination of active intermediates for elucidating the reaction mechanism. *Insitu* and *operando* ssNMR techniques have been used for the detection of active intermediates formed during catalytic reactions [18,112–114]. *Insitu* ssNMR often refers to the experiments performed under batch conditions, allowing the reactions to be measured under the state relevant to the real reaction [115]. For example, the catalysts and reactants are sealed in a glass ampoule reactor, the reactions are performed outside the NMR magnet and the ‘fossilized’ species on the catalyst are measured at room temperature after the reactions are intermittently quenched, usually using liquid N_2_. Therefore, even the short-lived intermediates formed in fast reactions can be trapped on the catalyst and analysed using NMR. Besides, the reactions can be conducted at a wide range of temperatures and pressures, depending on the glass ampoule reactor applied. However, neither adding reactants nor eliminating reaction products is possible during the reaction. *Operando* ssNMR allows continuous NMR measurements during the catalytic reaction under flow conditions in the NMR probe [116]. This method can be used to monitor the real reaction process resembling that in the fixed-bed reactor. Since a large number of signal accumulations is often required, it is difficult to follow fast reactions. Additionally, the reaction is usually limited to atmospheric pressure because the NMR rotor is not perfectly closed for the flow of the reactant. In this section, the most recent progress on the *insitu*/*operando* ssNMR study of zeolite-catalysed reactions is discussed with an emphasis on methanol and ethanol conversions.

**Figure 10.**
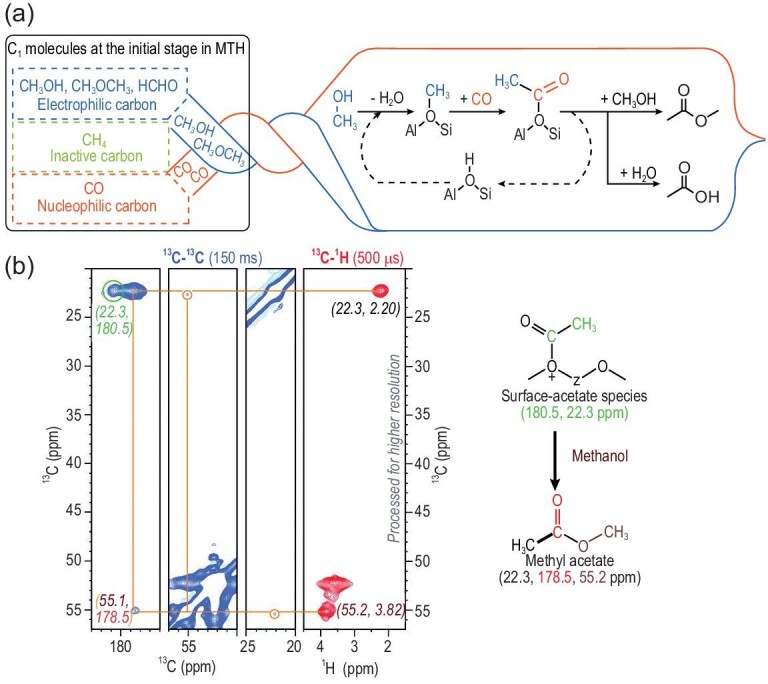
(a) Schematic of first C–C bond formation through coupling between surface methoxy group and CO. Adapted from [125] with permission from Wiley-VCH. (b) 2D ^13^C–^13^C PDSD (blue) and ^13^C–^1^H HETCOR (red) NMR spectra of H-SAPO-34 after the MTO reaction, indicating surface-acetate and methyl-acetate resonances. Adapted from [127] with permission from Wiley-VCH.

The MTO process on acidic zeolites has attracted special attention due to its potential to provide a non-petrochemical route for light olefins production [117]. The MTO process is a complex reaction network involving methylation, alkylation, oligomerization and cracking, etc., which makes a definite elucidation of the reaction rather difficult. The formation of the initial C–C bond in the MTO reaction has a great implication in the methanol chemistry, although the exact route remains a matter of considerable debate [118–122] partially due to the lack of solid experimental evidence for the key intermediates involved. ssNMR spectroscopy contributes to a deeper understanding of the C–C bond formation mechanism in the MTO reaction. By using the continuous-flow ^13^C NMR technique, Liu *et al.* observed the interactions between dimethyl ether (DME) species and surface methoxy species/trimethyloxonium ion species on ZSM-5 at the very beginning of the MTO process under *operando* conditions [123]. The highly polarized C–H bond in the DME was proposed to form a methyleneoxy-analog species (CH_3_–O–CH_2_–H–zeolite), which could serve as an activated C1 species (DME) for the first C–C bond formation. A different route for the activation of DME was proposed by other researchers [124]: DME firstly activated by surface methoxy species to form a methoxymethyl cation (CH_3_OCH_2_^+^) intermediate, which subsequently interacted with another DME molecule to form C–C bond-containing compounds 1,2-dimethoxyethane. Recently, the work of Lercher *et al.* indicated that surface methoxy species produced from methanol (DME) could couple with CO forming C–C bond species such as acetic acid and methyl acetate (Fig. 10a) [125]. The formation of methyl acetate via a surface-acetate intermediate has been ascertained in the *insitu*^13^C NMR study of DME carbonylation with CO over a mordenite zeolite [126]. By using 2D ^13^C–^13^C and ^1^H–^13^C NMR experiments, the surface-acetate species (180.5, 22.3 ppm) and methyl acetate (22.3, 178.5, 55.2 ppm) were recently identified in the MTO reaction over H-SAPO-34 [127] (Fig. 10b). These species were explained by the carbonylation reaction between CO and surface methoxy species as proposed on the H-ZSM-5 zeolite [125]. Light olefins (C_2_^=^–C_4_^=^) could be subsequently produced from these C–C bond species via the formation of ketene and derivatives [128].

**Figure 11.**
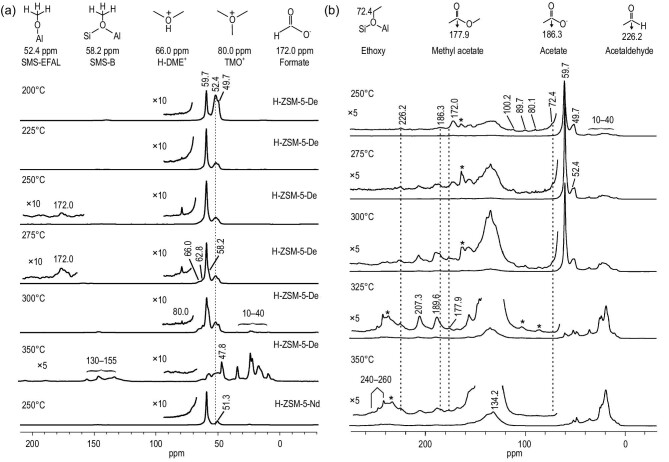
(a) ^13^C CP/MAS NMR spectra of trapped products obtained from reaction of ^13^C-methanol over ZSM-5 at different temperatures for 1 min and (b) ^13^C CP/MAS NMR spectra of trapped products obtained from reaction of ^13^C-methanol, followed by co-feeding ^13^C-methanol and ^13^C-formaldehyde over H-ZSM-5-De zeolite at 250–350°C. Adapted from [129] with permission from Wiley-VCH.

EFAL species lend Lewis acidity and a distinct reaction property to zeolites. Most recently, the critical role of EFAL species in mediating the first C–C bond formation was revealed in zeolites [129]. ^13^C MAS NMR spectra of the dealuminated zeolite (H-ZSM-5-De) indicated the formation of surface methoxy species bound to EFAL (SMS-EFAL) (52.4 ppm), which was unobservable on a non-dealuminated sample (H-ZSM-5-Nd) (Fig. 11a). This SMS-EFAL species was structurally confirmed using a ^13^C–{^27^Al} double-resonance NMR experiment. Further methanol and formaldehyde co-reaction experiments confirmed that this SMS-EFAL species can initiate the formation of several C–C bond species including acetaldehyde, ethanol and surface ethoxy species, and eventually ethene products (Fig. 11b). DFT calculations were used to optimize the elementary steps involved in the C–C bond formation. The activation energy barrier of the rate-limiting step in the C–C bond formation pathway initiated by the SMS-EFAL species was determined to be ∼30 kcal mol^–1^, which is much lower than that by the BAS (∼49 kcal mol^–1^). This work provided the first spectroscopic evidence of the formation of ethoxy species via the SMS-EFAL species and formaldehyde in the MTO reaction, which has been generally proposed as the precursor to the initial C–C bond formation in the direct mechanism [119,130]. Acidic zeolites could share a common property for the initial C–C bond formation in the MTO reaction. This was demonstrated over the H-SSZ-13 zeolite, on which the surface ethoxy species was *insitu* captured in the early reaction stage of methanol conversion by using the *insitu*^13^C NMR method [131].

**Figure 12.**
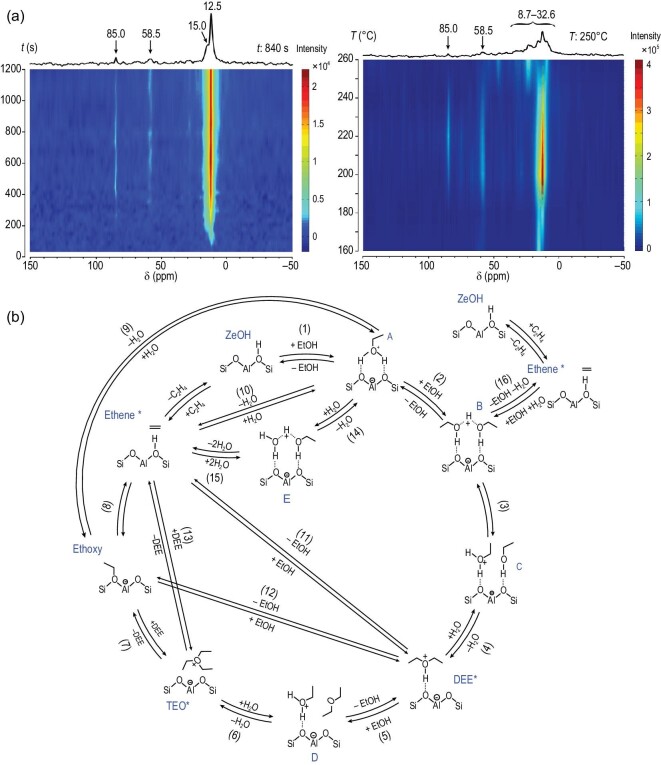
(a) *Operando*^13^C MAS NMR spectra of ^13^CH_3_^13^CH_2_OH dehydration on H-ZSM-5 as a function of reaction time and temperatures and (b) proposed catalytic cycle for ethanol dehydration to ethene. Adapted from [135] with permission from the Nature Publishing Group.

As a ‘sister-reaction’ of methanol to hydrocarbons (MTH), conversion of ethanol to hydrocarbons (ETH) has attracted increasing attention from both academia and industry because of the large availability of bioethanol from renewable biomass sources [132]. The first step of ethanol conversion on solid acids is dehydration to ethene followed by secondary reactions of polymerization, cracking and aromatization [133], in a similar way to the MTH reaction over zeolites [120]. *Insitu*^13^C NMR with UV-Vis spectroscopy has been used to investigate the ethanol dehydration process over Y zeolites [134]. The surface ethoxy species was unambiguously identified to be the key intermediate responsible for the dehydration of ethanol and the further transformation into higher hydrocarbons. The formation of ethoxy species is of research interest due to its importance in ethanol conversion. In a recent report [135], ethanol dehydration over zeolites was investigated under continuous-flow conditions using the *operando* NMR technique. A triethyloxonium ion (TEO) (85 ppm) was observed during ethanol dehydration on ZSM-5 (Fig. 12a). This species exhibited high reactivity during the reaction. It was decomposed at elevated temperature with concurrent formation of higher hydrocarbons (8.7–32.6 ppm) (Fig. 12b). TEO species was supposed to be generated from the dehydration of three ethanol molecules, showing similarity to the formation of a trimethyloxonium ion (TMO) in the methanol reaction in zeolites [136]. ^13^C NMR experiments indicated that TEO can be easily transformed to surface ethoxy species and then ethene. Among a complex ethanol dehydration network, the TEO–ethoxy route was theoretically identified as the most favorable route for ethene formation (Steps 1–8 in Fig. 12c).

**Figure 13.**
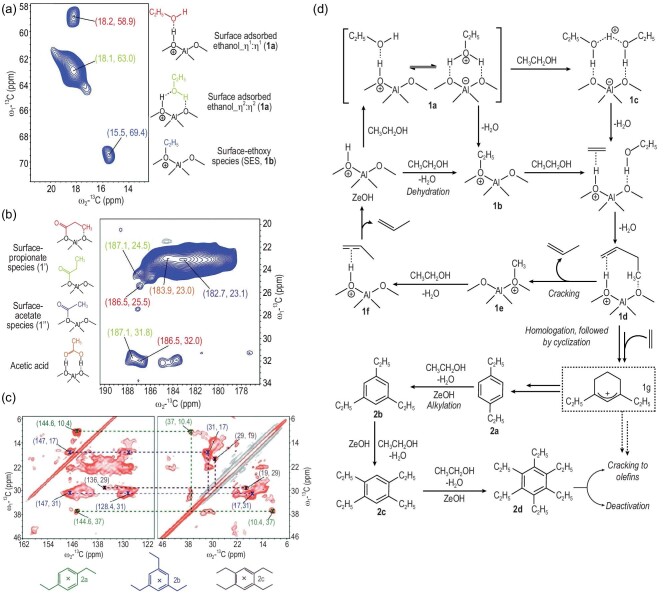
(a) Magnification in the surface-adsorbed alkoxy region and (b) in the carbonyl region of the 2D ^13^C–^13^C NMR spectra of trapped molecules in zeolite; (c) 2D ^13^C–^13^C NMR correlation NMR spectra of ethylated aromatics trapped in zeolite and (d) proposed mechanism for the homologation-reaction-dominated carbon−carbon bond coupling routes during the zeolite-catalysed ETH process. Adapted from [137] with permission from Wiley-VCH.

Higher hydrocarbons are readily produced after ethanol dehydration. Weckhuysen *et al.* investigated the exact mechanistic routes to the HP species in the reaction of ethanol dehydration on zeolites by using ssNMR and UV-Vis diffuse reflectance spectroscopy [137]. A series of adsorbed species over ZSM-5 after ETH reactions were identified in the 2D ^13^C–^13^C NMR spectra (Fig. 13a–c). Besides the adsorbed ethanol and surface ethoxy species, carbonylated surface species were observed on the zeolite, indicating the presence of similar Koch-carbonylation-based C–C bond-forming reactions as discussed in the above methanol conversion. The identification of ethylated aromatics trapped in zeolites suggested the prevalence of homologation reactions in the HP process. In the proposed reaction mechanism, the formation of ethene by ethanol dehydration via surface ethoxy species initiated the homologation reaction to produce butylene and non-homologation reaction to propylene (Fig. 13d). The dominating homologation reaction was responsible for the formation of olefins and ethylated aromatics in the ETH process. Since the ethylated aromatics are the main HP species in the ETH reaction, the deficiency of the olefins cycle makes the ETH process different from the MTH reaction, which involves both olefins- and aromatics-based cycles [138]. The active intermediate such as cyclic carbenium species that was proposed to be involved in the formation of aromatics in the ETH process was experimentally identified using ^13^C NMR spectroscopy [139]. Cyclopentenyl cations were observed during the ETH reaction on the ZSM-5 zeolite. These intermediates were closely related not only to aromatics, but also to propene products.

## CONCLUSION AND PROSPECTS

We have discussed the recent advances in the characterization of zeolites by using ssNMR spectroscopy. Combined with advanced instrumentation and experimental techniques, ssNMR has been demonstrated to be a powerful analytic tool in zeolites characterization. The direct detection of framework structures and acid sites is enabled by using various 1D and 2D ssNMR methods. The joint use of ssNMR with other techniques such as UV–Vis spectroscopy of Co^2+^ cations and theoretical analysis provides a versatile strategy for the investigation of Al organization in zeolite frameworks [48], which were found to be controlled by synthesis conditions. The obtained knowledge has made it possible for zeolite scientists to optimize zeolites with improved catalytic performance in many important reactions such as methanol conversion, cracking of hydrocarbons and oligomerization of alkenes by tuning the Al siting and distribution in zeolite frameworks [25,29,140,141]. 2D correlation spectroscopy allows ssNMR to probe the internuclear spatial proximities and connectivities, which are associated with host–guest and guest–guest interactions in zeolites. Beyond the chemical properties, the host–guest interactions that occur in zeolites significantly influence the physical properties such as quantum size effects of the guest species. The understanding of various interactions in zeolites allows the exploitation of optical, electronic and magnetic functions of zeolites [142]. The utilization of ssNMR for the observation and identification of critical active intermediates in zeolite-catalysed reactions has become a key approach for the elucidation of reaction mechanisms. The knowledge on the reaction mechanisms and the involved intermediates has been applied to the synthesis of new zeolites capable of controlling reaction pathways in a complex reaction such as methanol conversion [143].

Although most of the introduced NMR methods have been routinely applied and tremendous progress has been achieved, considerable challenges remain in zeolite chemistry for ssNMR characterization. One out of many examples is the high-resolution detection of dilute species or low content surface/interface species, which is fundamentally important in heterogeneous catalysis. The intrinsic low sensitivity of ssNMR hinders its application for this aspect, especially for the species involving infamous nuclei with low natural abundances and low γ features. Moreover, the complexity of zeolites, including diversified framework structure, heterogeneous distribution of the framework or extra-framework species and various covalent and non-covalent interactions, leaves a huge space for advanced ssNMR techniques. As an example, the full determination of crystallographically inequivalent T sites in ZSM-5 remains to be achieved in the application of the NMR technique to zeolites.

Further improvement can be expected from the development of ssNMR hardware and methodologies. The increasing availability of cutting-edge instrumentation such as ultra-high-field magnets and cryoprobes will promote ssNMR to a new level in terms of detection sensitivity and spectral resolution. On the other hand, the hyperpolarization method is emerging as a promising way to strengthen the application of ssNMR in material science. Particularly, the surface-enhanced DNP can provide a maximum 2–3 orders of magnitude sensitivity gain for the surface species, which turns into at least 4 orders of magnitude in experimental time-saving. The development of DNP methodology (e.g., radical formulation, sample preparation protocol) will fully unfold its potential in the characterization of zeolites and heterogenous catalysts in general. Another prospect of ssNMR in zeolites characterization is its combination with complementary methods such as XRD, UV-Vis, microscopy and computational simulation. With more detailed structure constraints and molecular dynamic parameters at different lengths and timescales, a clear picture of zeolite structures and related systems can be achieved.
